# *ATP-dependent DNA helicase* (*TaDHL*), a Novel Reduced-Height (*Rht*) Gene in Wheat

**DOI:** 10.3390/genes13060979

**Published:** 2022-05-30

**Authors:** Baojin Guo, Xuemei Jin, Jingchuan Chen, Huiyan Xu, Mingxia Zhang, Xing Lu, Rugang Wu, Yan Zhao, Ying Guo, Yanrong An, Sishen Li

**Affiliations:** 1 State Key Laboratory of Crop Biology, Tai’an 271018, China; guobaojin1991@126.com (B.G.); jinxm1124@163.com (X.J.); 13608988364@163.com (J.C.); xvhuiy@163.com (H.X.); zhangmingxia0506@163.com (M.Z.); luxing3886@163.com (X.L.); zhaoyan@sdau.edu.cn (Y.Z.); guoying729@126.com (Y.G.); anyanrong2002@163.com (Y.A.); 2 College of Agronomy, Shandong Agricultural University, Tai’an 271018, China; 3 Rizhao Academy of Agricultural Science, Rizhao 276826, China; 4 Dezhou Academy of Agricultural Science, Dezhou 253015, China; w882002@126.com

**Keywords:** wheat, plant height, *ATP-dependent DNA helicase* (*TaDHL*), CRISPR/Cas9

## Abstract

In wheat, a series of dwarf and semi-dwarf plant varieties have been developed and utilized worldwide since the 1960s and caused the ‘Green Revolution’. To date, 25 reduced-height (*Rht*) genes have been identified, but only several genes for plant height (PH) have been isolated previously. In this study, we identified a candidate gene, *ATP-dependent DNA helicase* (*TaDHL-7B*), for PH via QTL mapping and genome-wide association study (GWAS) methods. We knocked out this gene using the CRISPR/Cas9 system in variety ‘Fielder’. Two homozygous mutant genotypes, *AAbbDD* (−5 bp) and *AAbbDD* (−1 bp), were obtained in the T_2_ generation. The PH values of *AAbbDD* (−5 bp) and *AAbbDD* (−1 bp) were significantly reduced compared with the wild-type (WT, ‘Fielder’), indicating that *TaDHL-7B* is a novel *Rht* gene that controls the PH. This is the first time that a PH gene of wheat has been isolated with a non-hormone pathway, providing a new insight into the genetic control of PH. The *TaDHL* gene reduced the PH without a yield penalty. It could be used to improve the lodging resistance and yield in wheat breeding programs.

## 1. Introduction

Wheat (*Triticum aestivum* L.) is one of the most important cereal crops in the world and provides about one fifth of the calories in food for human consumption [1]. Optimizing a plant’s stature to enhance productivity is one of the major strategies of plant breeders [2]. Dwarf plants possess short, strong stalks and do not lodge, which could partition a greater proportion of assimilates into the grain and increase grain yield [3,4,5]. In wheat, a series of dwarf and semi-dwarf plant varieties have been developed and utilized worldwide since the 1960s and caused the ‘Green Revolution’ [6,7,8]. It is, thus, of great significance to identify novel dwarfing genes in wheat.

To date, 25 reduced-height (*Rht*) genes have been identified in wheat, distributed on chromosomes 2A, 2B, 2D, 3B, 4B, 4D, 5A, 5D, 6A, 7A, and 7B [9,10,11]. However, only the ‘Green Revolution’ genes *Rht-B1b*, *Rht-D1b*, *Rht8*, and *Rht9* have been utilized worldwide in wheat breeding [12]. The *RhtB1b* and *RhtD1b* genes in ‘Norin 10′ were derived from the Japanese semi-dwarf variety ‘Daruma’, and a large number of semi-dwarf wheat varieties have been developed from ‘Norin 10′ [13]. Another ‘Green Revolution’ gene, *Rht8*, was derived from the Japanese variety ‘Akakomugi’ and was introduced into Southern European wheat varieties in the 1930s by the Italian breeder Nazareno Strampelli, together with the *Ppd-D1a* allele [8]. The *Rht8* gene is well-adapted to water-limited conditions as it benefits from early seedling vigor and a longer coleoptile [14]. Some dwarfing genes, such as *RhtB1b*, *Rht-D1b*, *Rht11*, and *Rht17*, have adverse effects, such as a reduction in the coleoptile length, early vigor, and root length [14]. The *Rht5*, *Rht12*, *Rht13*, and *Rht18* genes reduce height and decrease grain weight [15,16].

Only a limited number of genes for plant height (PH) have been isolated previously. The *RhtB1b* (*Rht1*) and *RhtD1b* (*Rht2*) genes were cloned in 1999 [6] and encode the DELLA protein, a negative transcriptional regulator of the gibberellin signaling pathway [17]. The two genes and their dwarfing alleles, *Rht-B1c* (*Rht3*), *Rht-D1c* (*Rht10*), *Rht-B1e* (*Rht11*), and *RhtB1p* (*Rht17*), are GA-insensitive, while the other *Rht* genes are responsive to GA (GA-sensitive) [10]. Recently, the gene of *Rht8*, *TraesCSU02G024900* or *TraesCSU03G0022100*, was isolated, which encodes a ribonuclease H-like protein that regulates plant height via participating in GA biosynthesis regulation [18,19]. The causal gene of *Rht24*, *TraesCS6A02G221900* (*TaGA2ox-A9*), was also isolated, which encodes a gibberellin (GA)2-oxidase. The *Rht24b* allele was found to confer a higher expression of *TaGA2ox-A9* in stems, leading to a reduction in bioactive GA in stems but an elevation in leaves with no yield penalty [20]. A novel gene, *WUS CHEL-related homeobox-like* (*TaWUS-like*), was identified that regulates the sheathed spike and plant architecture. The *TaWUS-like* gene may inhibit the synthesis of GA_3_ and/or brassinosteroids (BRs), thus affecting the function of signal transduction, which further causes stem shortening and plant dwarfing [21].

Gene editing is a powerful tool for studying gene functions and can be used to manipulate the genome precisely and efficiently. Li et al. (2022) used gene editing to describe *Tamlo-R32*, a wheat *mlo* mutant that confers robust disease resistance without undesirable pleiotropic effects [22]. Song et al. (2022) identified a 54-base-pair cis-regulatory region in *IPA1* via a tiling-deletion-based CRISPR/Cas9 screen and resolved the tradeoff between grains per panicle and tiller number, leading to a substantially enhanced grain yield per plant [23].

In this study, we identified a candidate gene, *ATP-dependent DNA helicase* (*TaDHL*), for PH via QTL mapping and genome-wide association study (GWAS) methods. We validated the function of the *TaDHL* gene using a CRISPR/Cas9 system. The results of this study should provide further insights into the genetic control of PH and enrich the genetic resources for dwarf breeding in wheat.

## 2. Materials and Methods

### 2.1. Plant Materials and Trial Design

QTL analysis was performed on a set of 184 recombinant inbred lines (RILs) derived from a cross of ‘TN18 × LM6′ (TL-RILs, F_11_ in the 2015–2016 growing season) by single-seed descent (SSD) [24]. ‘TN18′ is a cultivated variety that was developed by our research group, while ‘LM6′ is an excellent line that was developed by the Linyi Academy of Agricultural Sciences, Shandong, China. The field trial and the mineral element trial were conducted at the experimental station of the Shandong Agricultural University (Tai’an, China) (Supplementary Table S1). For the field trial, seeds were sown on October 5–10, and plants were harvested on June 10–15 of the next year. Each plot consisted of three rows, which were 1.5 m long and spaced 25 cm apart, with two repetitions. Fifty seeds were planted in each row. The mineral element trial included four treatments (control, low N, low P, and low K) [25,26]. For the GWAS, we used an association population composed of 272 current wheat varieties from the Huang-huai Winter Wheat Region of China [27]. The experimental fields were located at the experimental station of the Shandong Agricultural University (Tai’an, China), the Rizhao Academy of Agricultural Sciences (Rizao, China), and the Dezhou Academy of Agricultural Sciences (Dezhou, China) in the 2019–2020 and 2020–2021 growing seasons and kept under watered and rainfed conditions.

### 2.2. QTL Analysis of the TL-RILs

A UPG-Map (a genetic map of unigenes based on their physical positions) for the TL-RILs was constructed [24] that includes 27,452 loci, 28,811 unigenes, 31,445 sub-unigenes, and 117,758 SNPs/InDels. Using the UPG-Map and phenotypic data, we carried out QTL analysis using IciMapping 4.1 (http://www.isbreeding.net/ accessed on 28 January 2019), Windows QTL Cartographer 2.5 (http://statgen.ncsu.edu/qtlcart/WQTLCart.htm accessed on 1 August 2019), and MapQTL 5.0 (https://www.kyazma.nl/index.php/mc.MapQTL/ accessed on 1 August 2019) [28] software. For Windows QTL Cartographer 2.5, composite-interval mapping (CIM) was used to search for QTLs with a walk speed of 0.5 cM. For IciMapping 4.1, inclusive composite-interval mapping (ICIM) [29] was carried out in steps of 0.5 cM. For MapQTL 5.0, the multiple-QTL model (MQM) package with a mapping step size of 0.5 cM was used to map the QTLs.

### 2.3. GWAS for the Association Population

We had previously carried out the RNA sequencing of the 272 varieties and identified 176,357 polymorphic SNPs/InDels (including 163,223 SNPs and 13,134 InDels) [27]. Based on phenotype and these markers, GWAS was performed using TASSEL version 5.0 with a mixed linear model that took into account kinship and PCA [30] and GAPIT version 3.0 with a compressed mixed linear model (CMLM) [31].

### 2.4. DNA and RNA Extraction for ‘Fielder’ and Mutant Plants

For variety ‘Fielder’ (wild-type, WT) and the progenies of gene editing, total DNA was extracted using a DNA extraction kit (Tiangen, Beijing, China). The quality and concentration of the total DNA were determined using a NanoDrop 2000c spectrophotometer (Thermo, Wilmington, DE, USA). Total RNA was extracted using the RNAprep Pure Plant Kit (TIAGEN, Beijing, China). The RNA’s purity was checked using a nanophotometer spectrophotometer (IMPLEN, CA, USA). The RNA’s integrity was assessed using the RNA Nano 6000 Assay Kit for the Agilent Bioanalyzer 2100 system (Agilent Technologies, CA, USA). Reverse-transcription into cDNA was performed according to the manufacturer’s protocol.

### 2.5. Gene Editing Using the CRISPR/Cas9 System

Total DNA was used to amplify *TaDHL-7B* (*TraesCS7B02G055300*) in ‘Fielder’. The primers were *TaDHL*-*F*: CCTCTCTCGAATCATTCGCC and *TaDHL*-*R*: TGAAGAAGGAACCTAAATG. To identify the function of *TaDHL*-*7B* in wheat, we knocked out this gene using the CRISPR/Cas9 system. An sgRNA targeting a conserved region within the second exon was designed in CRISPR-direct (http://crispr.dbcls.jp/ accessed on 13 January 2020) (guideRNA, CGTAGATGACTGCCCTGCATCGG). The CRISPR/Cas9 vector pLGYE-3 was constructed and then transformed into ‘Fielder’. Gene editing was conducted by the Crop Research Institute at the Shandong Academy of Agricultural Sciences [32]. The sequences of edited plants were analyzed using Hi-TOM analysis [33]. The specific primers used to detect mutations of *TaDHL* in the Hi-TOM platform were *Hitom-F*: ggagtgagtacggtgtgcTAGCCCATTGGGTCTTCG and *Hitom-R:* gagttggatgctggatggCTTACCTCCCATTTGCCT. The knock-out mutants were planted in pools and pots in a greenhouse.

### 2.6. qRT-PCR, Sequence Characteristics, and Bioinformatics Analysis

The transcript levels of *TaDHL*-*7B* in ‘Fielder’ organs were analyzed by qRT-PCR. The first-strand cDNAs were synthesized by 2 μg of RNA per sample using TransScript One-Step gDNA Removal and cDNA Synthesis SuperMix (TransGen, Beijing, China). The qRT-PCR amplifications were performed as described by Zhao et al. [34]. Amplification of *TaActin* was used as an internal control for data normalization. The experiments were independently replicated three times under identical conditions. The complete alignment of multiple coding sequences and translations of nucleotides into amino acid sequences were performed using the DNAMAN program (Version5.2.2, Lynnon Bio-soft, Canada). The hydrophilic/hydrophobic analysis was performed using Prot Param (https://web.expasy.org/protparam/, accessed on 1 May 2022). The prediction of the transmembrane structure was performed using DeepTMHMM (https://dtu.biolib.com/DeepTMHMM, accessed on 14 April 2022). The collinearity analysis was performed using Triticeae-Gene Tribe (TGT, http://wheat.cau.edu.cn/TGT/m.html?navbar=MicroCollinearity, accessed on 1 May 2022). The prediction of the three-dimensional structures of proteins was performed using Swiss-model (https://swissmodel.expasy.org/interactive, accessed on 1 May 2022).

## 3. Results

### 3.1. Acquisition of the Candidate Gene for PH

**Figure 1:**
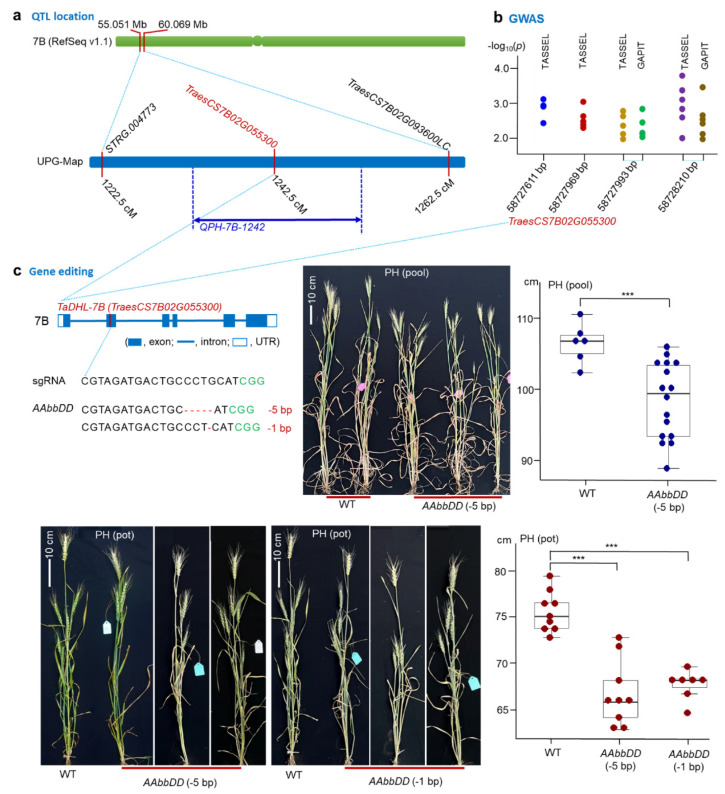
Physical region, QTL location, GWAS, and gene editing of *TaDHL7B*. (**a**) QTL location for *QPh-7B-1242* determined using TL-RILs and their UPG-Map. The candidate gene *TraesCS7B02G055300* was identified. (**b**) GWAS using an association population and its polymorphic SNPs/InDels. (**c**) Gene editing of *TaDHL-7B* using the CRISPR/Cas9 system, indicating that *TaDHL-7B* controls the PH. *** *p* < 0.001.

Combining the UPG-Map of the TL-RILs and phenotypic data under multiple environments, we obtained a stable QTL for PH, *QPh-7B-1242*, using three software programs, WinQTLCart 2.5, IciMapping 4.1, and MapQTL 5.0, simultaneously (Supplementary Table S1). The yield components, spike number per unit area (SN), grain number per spike (GNS), and thousand-grain weight (TGW) were not located on this QTL. The additive effects of *QPh-7B-1242* were positive, with ‘TN18′ increasing the PH (Supplementary Table S1). The peak positions of the QTL ranged from 1234.5 to 1252.5 cM, which covered only one candidate gene (*TraesCS7B02G055300*) (Figure 1a). This gene encoded an *ATP-dependent DNA helicase*, and we named it the *TaDHL*-*7B* gene. The *TaDHL-7B* gene had one SNP at 1451 bp (starting at ATG) in the exon with a nonsynonymous substitution (Arginine for ‘TN18′ to Tryptophan for ‘LM6′). These results indicate that *TaDHL-7B* was the candidate gene of *QPh-7B-1242*.

Using an association population of 272 current varieties, we performed a GWAS in multiple environments. Four SNPs/InDels of *TaDHL-7B*, 58,727,611 (A > AG), 58,727,969 (G > A), 58,727,993 (T > C), and 58,728,210 (T > C) bp in the RefSeq v1.1 genome [1], were significantly associated with PH at the level of *p* < 0.01 in multiple environments using the TASSEL 5.0 software. The latter two SNPs were also significantly associated with PH according to the GAPIT 3.0 software (Figure 1b and Supplementary Table S2). The four markers were not associated with yield components. These results also indicate that *TaDHL-7B* is the candidate gene of PH.

### 3.2. Confirming the Function of the TaDHL-7B Gene Using the CRISPR/Cas9 System

**Figure 2:**
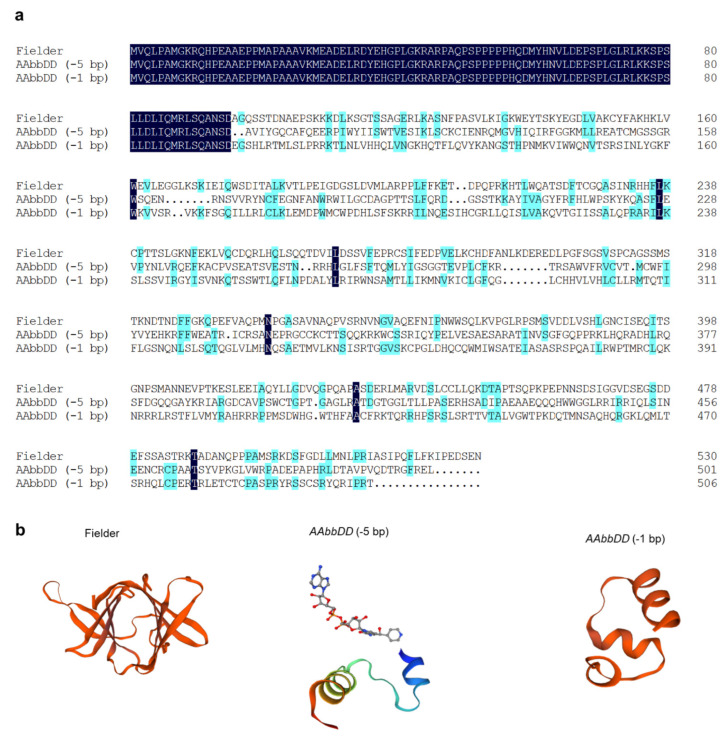
Amino acid sequences and prediction of the three-dimensional structures of the *TaDHL-7B* protein for ‘Fielder’ and the mutant genotypes *AAbbDD* (−5 bp) and *AAbbDD* (−1 bp). (**a**) Amino acid sequences; (**b**) three-dimensional structures of the protein.

We performed gene editing of sub-genome B of *TaDHL* using the CRISPR/Cas9 system (Figure 1c). In the T_2_ generation, two homozygous mutant genotypes, *AAbbDD* (−5 bp) and *AAbbDD* (−1 bp), were obtained. *AAbbDD* (−5 bp) deleted 5 bp at 285 bp (starting at ATG) and *AAbbDD* (−1 bp) deleted 1 bp at 286 bp in the second exon region of *TaDHL-7B* (Figure 1c, Figure 2a, and Figure S1). *AAbbDD* (−5 bp) encoded 102 amino acids and *AAbbDD* (−1 bp) encoded 122 amino acids. The two mutant genotypes lead to frameshift mutations and make the termination codon appear in advance, leading to functional inactivation of the protein (Figure 2a). The three-dimensional structures of proteins for *AAbbDD* (−5 bp) and *AAbbDD* (−1 bp) indicate that the structures are incomplete compared with that of the WT (Figure 2b).

We planted the WT and knock-out mutant plants of the T_2_ generation in pools and pots, respectively (Figure 1c and Supplementary Table S3). In the pool trial, the homozygous mutant genotype *AAbbDD* (−5 bp) was found in 16 plants. The PH values of the WT and *AAbbDD* (−5 bp) were 106.6 and 98.6 cm, respectively, with significant differences between them. The PH of *AAbbDD* (−5 bp) was 8.0 cm less than that of the WT. In the pot trial, the two homozygous mutant genotypes *AAbbDD* (−5 bp) and *AAbbDD* (−1 bp) were found within nine and seven plants, respectively. The PH values of the WT, *AAbbDD* (−5 bp), and *AAbbDD* (−1 bp) were 75.7, 66.8, and 67.8 cm, respectively, with significant differences between them. The PH of *AAbbDD* (−5 bp) and *AAbbDD* (−1 bp) had been reduced by 8.9 and 7.9 cm compared with the WT. These results indicate that the *TaDHL-7B* gene controls the PH.

### 3.3. Characteristics of TaDHL-7B

**Figure 3:**
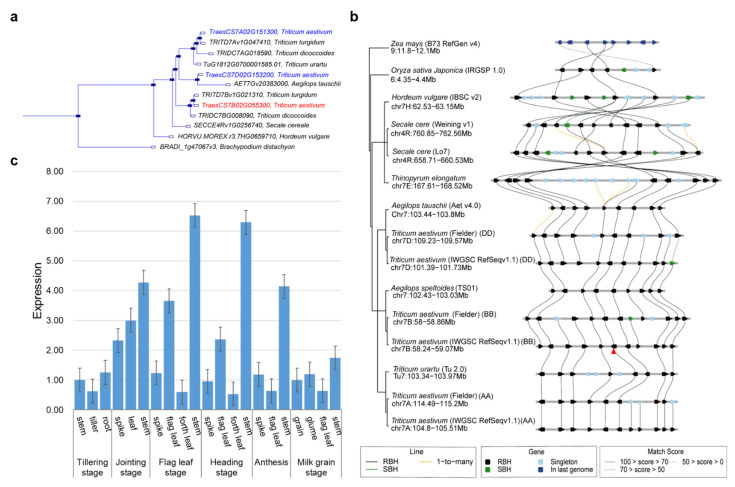
Characteristics of *TaDHL-7B*. (**a**) Phylogenetic tree for *TaDHL-7B.* (**b**) Collinearity analysis. (**c**) Expression levels of *TaDHL-7B* in different tissues and periods. Red triangle refers to the gene *TaDHL-7B* in subfigure (**b**).

The sequence analysis of the *TaDHL-7B* gene showed that the full length was 6125 bp with six exons and five introns and the coding sequence was 1593 bp and encoded 530 amino acids (Figure 1c and Figure S1). To investigate the evolutionary relationships, a phylogenetic tree of *TaDHL* proteins from various plant species was constructed. It showed that *TaDHL-7B, TaDHL-7A*, and *TaDHL-7D* were on different branches. The *TaDHL-7B* proteins were closely related to *TRITD7Bv1G021310* of *Triticum turgidum* and *TRIDC7BG008090* of *Triticum dicoccoides*; *TaDHL-7A* was closely related to *TRITD7Av1G047410* of *Triticum turgidum*; and *TaDHL-7D* was closely related to *AET7Gv2038300* of *Aegilops tauschii* (Figure 3a). To determine the collinearity relationship between related species, a collinearity analysis was performed and revealed that the *TaDHL-7B* region was conserved between genomes A, B, D, and E, but an inversion occurred in genome R (Figure 3b). The expression levels in different tissues and periods showed that *TaDHL-7B* is a constitutive gene, and the highest expression was found in the stem at the flag leaf stage and the heading stage (Figure 3c). The transmembrane structure prediction indicated that *TaDHL-7B* is not a transmembrane protein and it might be located inside the membrane (Supplementary Figure S2). These results indicate that *TaDHL-7B* is a constitutively expressed and relatively conserved gene.

## 4. Discussion

Wheat is an allohexaploid species with a large and highly complex genome that severely restricts the isolation of genes via map-based cloning. In this study, we identified only one candidate gene (*TaDHL-7B*) for the QTL *QPh-7B-1242* using QTL location and GWAS methods based on the RNA sequencing of each line of the RIL population and each variety of the association population, respectively. Then, we confirmed the function of *TaDHL-7B* using the CRISPR/Cas9 system. This should be an effective strategy for isolating genes originally from wheat.

Only a few genes for PH have previously been isolated, including *RhtB1b*, *RhtD1b*, *Rht8*, *Rht24*, and *TaWUS-like*. The first four genes reduce the PH via the GA pathway, and *TaWUS-like* may be related to the GA and/or BR pathway [6,18,19,20,21]. In this study, we isolated a novel gene, *TaDHL*, that controls PH. This is the first time that the *Rht* gene of wheat has been isolated with a non-hormone pathway, providing a new insight into the genetic control of PH. In addition, no yield components (SN, GNS, and TGW) were identified for *TaDHL* using QTL mapping and GWAS analysis, and no differences in the yield components were observed between mutant genotypes and the WT (Figure 1c), suggesting that *TaDHL* reduced the PH without a yield penalty. It may favor the improvement of the lodging resistance and yield in wheat breeding programs.

Helicases form a diverse group of molecular drivers and convert double-strand nucleic acids into single-strand nucleic acids with the help of ATP hydrolysis, thus facilitating the replication and transcription processes [35]. The DNA helicase plays an important role in DNA replication, DNA repair and recombination, chromosome segregation, and transcription initiation [36,37,38]. In humans, some DNA helicases play diverse roles in the response to DNA damage and have potential as novel cancer chemotherapeutic targets [39,40]. However, there is far less information about plant DNA helicases [41]. The first plant DNA helicase identified was from a lily [42]. Overexpression of a DNA helicase from *Pisum sativum* (PDH45) was found to improve the response to abiotic stresses, such as high salinity, cold stress, dehydration, and early wounding, without a yield loss [43]. In rice, suppression of *OsKu80* resulted in a reduction in growth at the post-germination stage and an increase in the telomere length [44]. We found that the function of *TaDHL-7B* was lost in knocked-out mutants, such as a weakening of the response to DNA damage, causing a decrease in plant height.

## 5. Conclusions

We identified a candidate gene for PH on the short arm of chromosome 7B via QTL mapping and GWAS analysis. This gene was annotated as an *ATP-dependent DNA helicase* (*TaDHL*) gene. We validated the function of the *TaDHL* gene using the CRISPR/Cas9 system, and knock-out *TaDHL-7B* mutant genotypes were found to significantly reduce plant height. *TaDHL-7B* is a novel *Rht* gene with a non-hormone pathway and without a yield penalty.

## Data Availability

Not applicable.

## Supplementary Materials

The following supporting information can be downloaded at: https://www.mdpi.com/article/10.3390/genes13060979/s1. Table S1: *QPh-7B-1242* for PH in the TL-RILs; Table S2: GWAS for PH using the association population of 272 wheat varieties; Table S3: PH for WT and mutant genotypes planted in pools and pots in the T_2_ generation; Figure S1: Coding sequences (CDS) for ‘TN18′, ‘LM6′, ‘Fielder’, and mutant genotypes *AAbbDD* (−5 bp) and *AAbbDD* (−1 bp); Figure S2: Prediction of the transmembrane structure of *TaDHL-7B*.
