# Supplementary material for: ATP-dependent DNA helicase (TaDHL), a Novel Reduced-Height (Rht) Gene in Wheat
Source: Genes (Basel). 2022 May 30;13(6):979. doi: 10.3390/genes13060979 (PMC9222645; doi:10.3390/genes13060979)
Supplement: Supplementary file 1 [file genes-13-00979-s001.zip › Supplementary Tables.pdf]

Table S1 *QPh-7B-1242* for PH in the TL-RILs

Software	Environment	Peak position(cM)	LOD	R^2 (%)	Additive effect
IciMapping 4.1	M(LKAV)	1234.5	2.15	11.43	1.12
	M(CK14)	1235.5	2.86	12.37	1.78
	M(LPAV)	1236.0	2.92	12.00	1.15
	F(E15)	1248.5	3.00	11.86	1.89
	F(E14)	1248.5	2.24	8.18	1.51
	M(LN16)	1250.0	2.17	8.05	1.44
	M(LNAV)	1250.5	3.06	12.98	1.40
Win QTL Cart 2.5	M(LPAV)	1242.5	2.08	4.19	0.74
	M(CKAV)	1243.8	3.19	5.32	1.10
	M(CK15)	1245.9	2.18	4.24	1.35
	M(LN16)	1246.8	2.23	5.05	1.35
MAPQTL 6.0	M(CK14)	1235.5	2.11	5.20	1.59
	M(CKAV)	1238.5	2.13	5.20	1.07
	M(LP16)	1241.5	2.02	4.90	1.06
	F(E11)	1252.5	2.59	6.30	1.75

F, Field trail; M, nutrient element trail.
CK, normal N, P and K; Ln, low N; LP, low P; LK, low K.
11, 2011; 14, 2014; 15, 2015; 16, 2016.

Table S2 GWAS for PH using the association population of 272 wheat varieties

Software	Environment	Position (RefSeq v1.1) (bp)	SNP/ InDel	*P value*	R^2 *(%)*	Gene
TASSEL	21TA-W	58727611	A > AG	1.37E-03	3.99	TraesCS7B02G055300
	21DZ-W			1.25E-03	4.08	
	21TA-D			8.77E-04	4.37	
	AV-W			1.26E-03	4.03	
	AV-D			3.66E-03	3.32	
	21TA-W	58727969	G > A	4.75E-03	3.15	TraesCS7B02G055300
	21DZ-W			3.91E-03	3.29	
	21TA-D			1.03E-03	4.30	
	AV-W			2.45E-03	3.59	
	AV-D			3.41E-03	3.39	
	21RZ-W	58727993	T > C	2.45E-03	3.62	TraesCS7B02G055300
	21DZ-W			6.94E-03	2.90	
	21TA-D			1.77E-03	3.90	
	AV-W			4.32E-03	3.19	
	AV-D			9.34E-03	2.67	
	20RZ-D	58728210	T > C	9.24E-03	2.69	TraesCS7B02G055300
	21TA-W			2.50E-03	3.61	
	21DZ-W			1.59E-03	3.94	
	21TA-D			2.09E-04	5.44	
	AV-W			8.85E-04	4.32	
	AV-D			5.04E-04	4.78	
GAPIT	21RZ-W	58727993	T > C	1.60E-03	18.08	TraesCS7B02G055300
	21TA-D			3.45E-03	10.34	
	21TA-W			6.44E-03	21.19	
	21RZ-D			7.13E-03	20.37	
	AV-W			8.51E-03	30.82	
	20RZ-D			8.64E-03	16.75	
	21TA-D	58728210	T > C	4.20E-04	10.34	TraesCS7B02G055300
	21TA-W			2.30E-03	21.19	
	AV-D			2.88E-03	32.81	
	20RZ-D			3.86E-03	16.75	
	21RZ-D			6.96E-03	20.37	
	AV-W			9.71E-03	30.82	

TA, Tai'an; RZ, Rizhao; DZ, Dezhou.
20, 2020; 21, 2021.
W, watered condition; R, rainfed condition.

Table S3 PH for WT and mutant genotypes planted in pool and pot in T_2 generation

Pool				Pot					
**Genotype**	**PH (cm)**	**Genotype**	**PH (cm)**	**Genotype**	**PH (cm)**	**Genotype**	**PH (cm)**	**Genotype**	**PH (cm)**
WT	107.2	*AAbbDD*	89.0	WT	78.1	*AAbbDD*	66.3	*AAbbDD*	66.8
	104.7	(-5 bp)	100.5		72.9	(-5 bp)	62.9	(-1 bp)	68.5
	110.6		102.5		76.7		68.2		68.2
	107.9		93.5		73.9		65.9		64.7
	106.5		96.5		75.2		71.9		69.7
	102.4		95.5		74.6		65.8		68.1
			104.0		73.8		64.2		68.3
			99.0		79.6		72.9		
			105.0		76.5		63.3		
			106.0						
			103.5						
			103.5						
			92.3						
			92.7						
			93.5						
			100.0						
AV	106.6		98.6	AV	75.7		66.8		67.8
