# Supplementary figures and images for: ATP-dependent DNA helicase (TaDHL), a Novel Reduced-Height (Rht) Gene in Wheat
Source: Genes (Basel). 2022 May 30;13(6):979. doi: 10.3390/genes13060979 (PMC9222645; doi:10.3390/genes13060979)

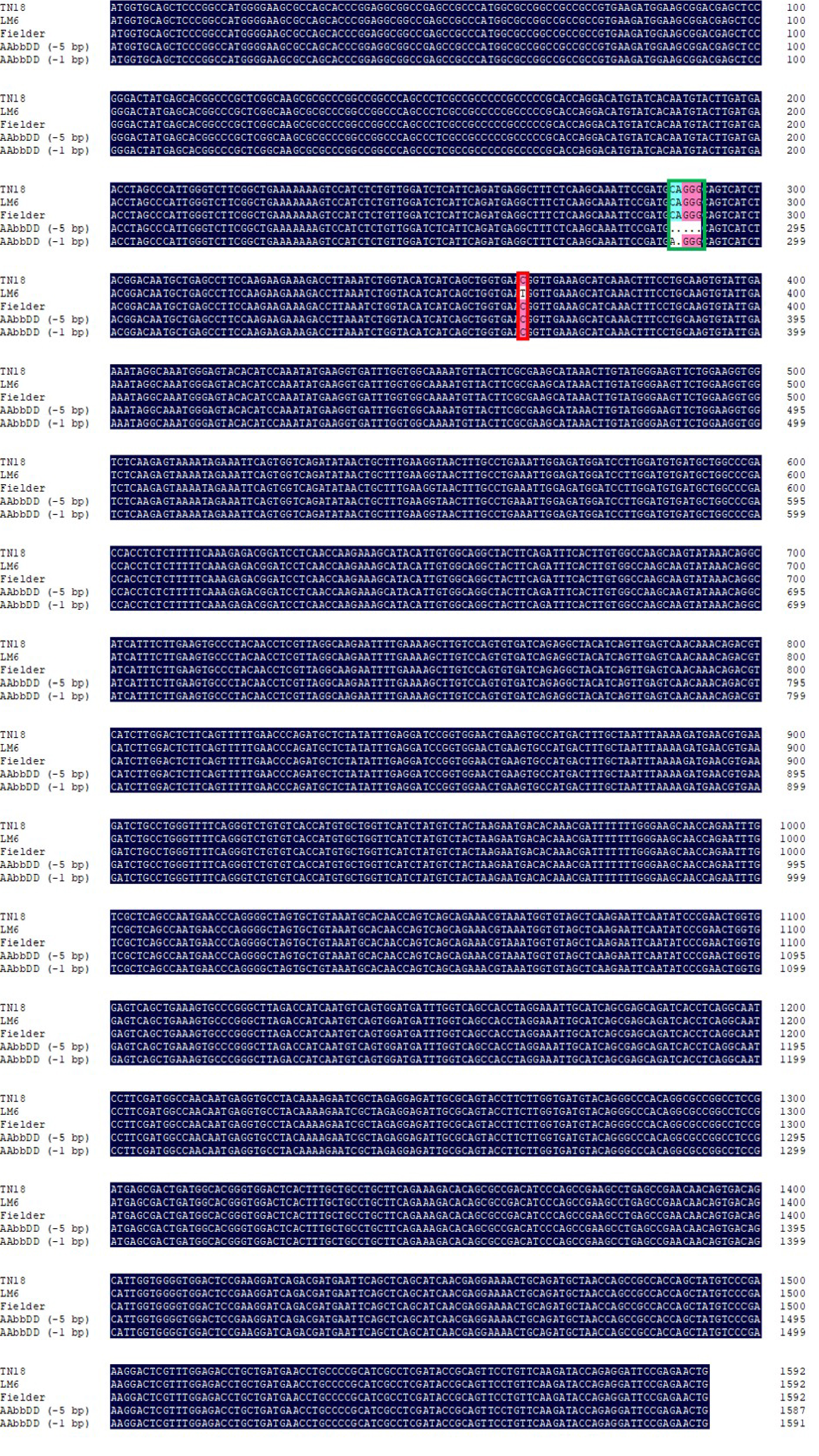

Supplement: Supplementary file 1 [file genes-13-00979-s001.zip › Figure S1.jpg]

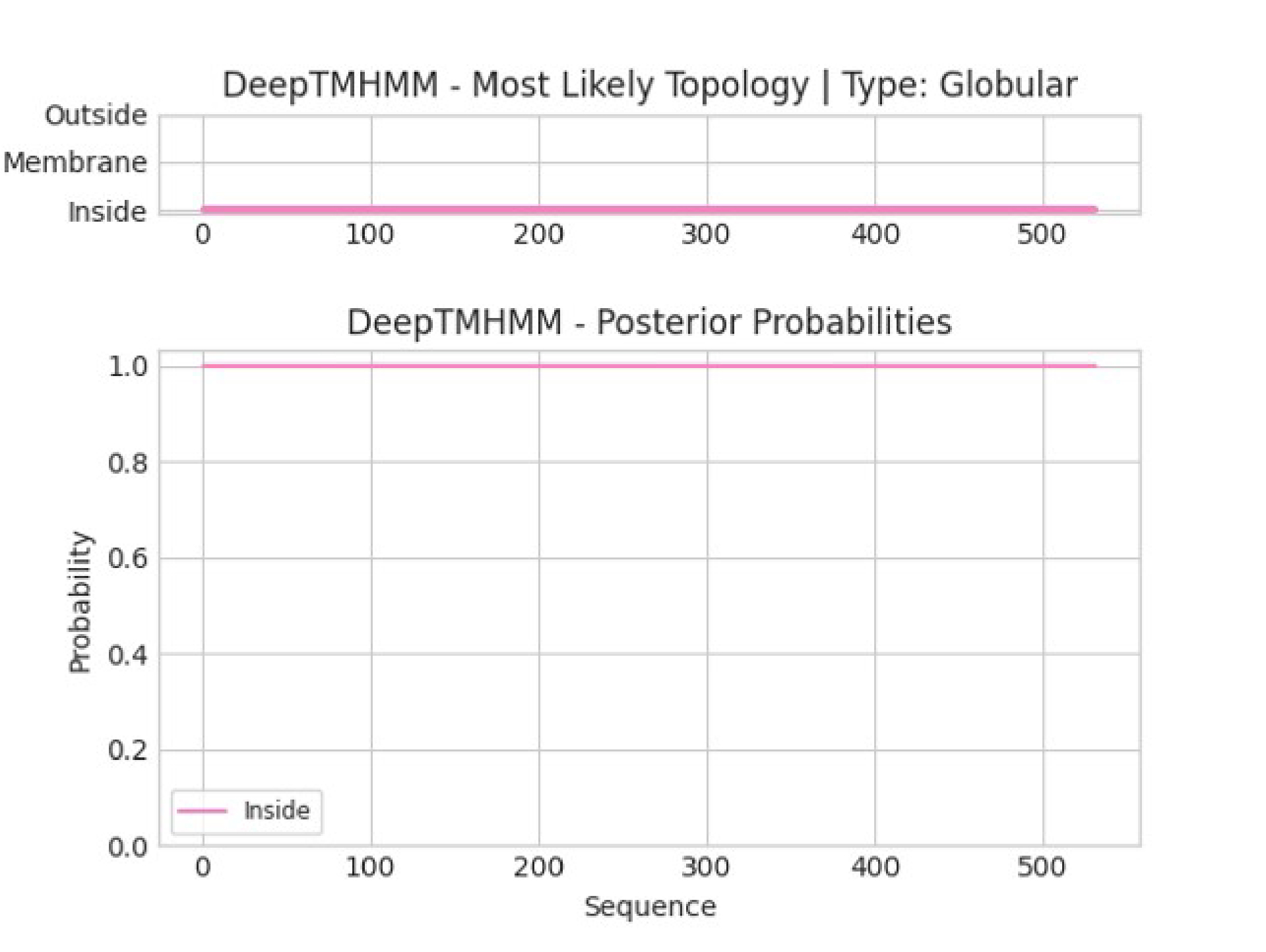

Supplement: Supplementary file 1 [file genes-13-00979-s001.zip › Figure S2.jpg]
